# Optimal Dietary Protein Requirement for Juvenile Sesarmid Crab (*Episesarma singaporense*)

**DOI:** 10.3390/ani10060998

**Published:** 2020-06-08

**Authors:** Chanyut Sudtongkong, Karun Thongprajukaew, Suktianchai Saekhow

**Affiliations:** 1 Department of Marine Science and Environment, Faculty of Sciences and Fisheries Technology, Rajamangala University of Technology Srivijaya, Trang 92150, Thailand; chanyuts@gmail.com; 2 Department of Applied Science, Faculty of Science, Prince of Songkla University, Songkhla 90112, Thailand; chaung_17@hotmail.com

**Keywords:** amino acid composition, dietary protein, digestive enzyme, protein efficiency ratio, sesarmid crab

## Abstract

**Simple Summary:**

Essential information on the major nutrients which could affect the survival and growth of early juvenile *Episesarma singaporense* crabs, especially their protein requirements, is still lacking. Therefore, the objective of this study was to determine the dietary protein requirement for early juvenile *E. singaporense* crabs. Growth performance, feed utilization, digestive enzyme activity, and muscle amino acid profiles were used as criteria for assessing the suitable treatment. Based on our investigations, desirable characteristics were achieved in the crabs fed with diet containing 45% protein. This level is similar to previous reports in other crab species, and could be employed in preparing artificial diets for this species.

**Abstract:**

The optimal dietary protein requirement for sesarmid crabs (*Episesarma singaporense*) was investigated. Juvenile *E. singaporense*, individually reared in plastic glasses containing 250 mL sea water, were fed five fish meal-soybean meal-microbound diets variously containing dietary protein levels of 30%, 35%, 40%, 45% and 50% for six weeks. A completely randomized design was used in the experiment, comprising five treatments with 60 crabs in each. At the end of the experiment, a significant improvement in survival was observed in all treatments relative to the diet containing 30% dietary protein (*p* < 0.05) while the growth performance parameters did not differ across the five dietary groups. A significantly higher protein efficiency ratio was observed in the *E. singaporense* crabs receiving 45% dietary protein relative to the remaining treatments. The specific activities of the digestive enzymes, pepsin-like, trypsin, amylase, and lipase, and the amylase to trypsin ratio fluctuated across the five treatments, but that of chymotrypsin remained consistent, suggesting different nutritional responses to the various dietary protein levels. The crabs receiving the 45% protein diet had significantly higher in essential amino acid (EAA) profiles followed by the 40% protein diet, although some EAA values were only moderate. The pattern for the non-EAA (NEAA) was reversed, and the ΣEAA/ΣNEAA ratio was higher in the crabs receiving the 45% protein diets relative to the other treatments. Based on our investigations, the optimal dietary protein requirement achieving desirable characteristics of juvenile *E. singaporense* crabs was 45%. This finding would be a useful guideline in preparing artificial diets for the mariculture of this species.

## 1. Introduction

*Episesarma singaporense* is a common species of sesarmid crab dwelt in the mangrove of the Malaysia peninsula [1]. This crab is one of five species in the *Episarma* genus, which are financially valuable fishery products. These crabs are gathered in enormous numbers for food in some South-East Asian nations and in some southern communities [2]. In Thailand, *E. singaporense*, together with *E. versicolor* and *E. mederi*, is gathered for consumption in customary Thai food. In view of Tiensongrassamee [3], 18,000 tons of sesarmid crabs (including *E. singaporense*) are caught from mangrove territories and consumed annually by the Thai people. However, 12,000 tons of crabs harvested annually from the Thai mangrove regions cannot fulfill domestic demand, and Thailand imports at least 6000 tons of sesarmid crabs from the neighboring countries of Myanmar and Cambodia. Therefore, it is important to increase awareness of sesarmid crab culture, in order to take measures to mitigate the scarcity of the species in Thailand. Macronutrient requirements are of particular interest to researchers focusing on boosting the survival and growth of aquatic animals. Nonetheless, important information is still required on key nutrients that could affect the survival and growth of the species, particularly their protein requirements.

Proteins are the dominant organic material in animal tissue, which therefore constitute significant dietary needed for aquatic animal growth [4,5]. Crustaceans (e.g., crabs) cannot synthesize all the amino acids they need and must therefore ingest protein or essential amino acids (EAA) through their dietary intake [6,7]. Determining protein requirements is often given priority in nutritional studies, because it is the costliest ingredient of crustacean dietary preparation [8,9] and plays a key role in growth [10,11]. Increasing protein levels in diets, especially for carnivorous animals, can generally lead to improved productivity [12]. Inadequate dietary protein contributes in reduced growth, as protein must be ingested from less essential tissues to sustain more vital ones [7]. However, if insufficient dietary protein is fed to animals, only a component is used to generate new growth tissues, with any surplus protein being metabolized as an energy source [12,13]. Excessive protein-containing diets not only result in increased amounts of energy used for excretion and increased production of nitrogen waste, but also in increased feed costs [12,13]. In addition, any reduction in dietary protein levels that does not negatively influence the growth of crustaceans will significantly reduce feed costs [9]. An understanding of protein requirements is therefore a critical factor in achieving cost-effective and sufficiently nutritious diets to ensure the optimal growth of crustaceans [11,14].

Optimal dietary protein levels of various species of crustaceans have already been recorded, with values ranging from 30 to 55% [9,10,15,16,17]. These levels of protein varied depending on several factors, such as species, size, age, digestibility and the amino acid composition of the diet [11]. Until now, the optimal protein level for early juvenile *E. singaporense* crabs has not been published. The aim of this study was therefore to determine the dietary protein requirement for early juvenile *E. singaporense*. Growth performance, feed utilization, digestive enzyme activity, and muscle amino acid profiles were used as criteria for assessing the suitable treatment. The knowledge gained from the study will contribute to the development of an artificial diet which is a key factor in the successful culture of this species.

## 2. Materials and Methods

Five diets were designed with a protein content of 30 to 50%. Fish meal, shrimp meal, soybean meal, and wheat gluten meal were used as sources of protein, while fish oil and soybean oil were the sources of lipids, and dextrin was the source of carbohydrates. Table 1 indicates the ingredients used in the five diets and their proximate chemical compositions. The amount of amino acids for each diet is shown in Table 2. The contents of fish oil and soybean oil were balanced to retain equal amounts of dietary energy, and the soy lecithin, mineral mixture, and vitamin mixture were maintained constant. By adding cellulose, the overall proportions were adjusted to 100%. The experimental diets were processed using the protocol of Holme et al. [18]. With an electric grinder (Model YB-2500A; Yongkang City Speed Feng Industry and Trade Co., Ltd., Zhejiang, China), all raw dry ingredients were finely ground and passed through a 0.425 mm mesh sieve. Subsequently, the dry ingredients were combined with wet ingredients (lipid sources and water), and then stirred to produce homogeneous dough. The resulting dough was extruded in a 3 mm-die diameter mechanical mincer (Model NG-12; Chaichana Partnership, Chanthaburi, Thailand) and dried overnight at 50 °C. After drying, the diet strands were cut in lengths of approximately 2 mm, wrapped in plastic bags and held in a freezer at −20 °C before they were used.

The composition of diets, such as moisture, crude protein, crude lipid, ash, and crude fiber, were analyzed in accordance with standard protocols of AOAC [19]. Nitrogen-free extract (NFE, %) and gross energy (GE, kcal kg^−1^) were determined from 100 − (moisture+crude protein+crude lipid+ash+crude fiber) and from (crude protein×5.6)+(crude lipid×9.44)+(crude fiber×4.1)+(NFE×4.1), respectively. Both chemical analyses were performed in triplicates, and are reported on a percentage of fresh weight.

Natural seawater was filtered using a 60 μm filter bag and stocked in a 10,000 L concrete tank for two days. To eliminate bacteria and other pathogenic microorganisms, the seawater was treated with 30 mg L^−1^ calcium hypochlorite for two days and then neutralized by strong aeration for 3 days. Afterward, the seawater was siphoned though the cartridge filters (10, 5 and 1 μm) into a storage plastic tank of 1000 L capacity. The treated seawater was used for broodstock, larval, and juvenile rearing, during which the water quality was controlled by aeration and exchange of water. Throughout this experiment, the water quality was monitored every 3 days. The pH, temperature, salinity, dissolved oxygen (DO), conductivity, and total dissolved solids (TDS) were measured by water quality meter (HoribaU-54 G; Horiba Instruments Limited, Kyoto, Japan). The analysis method described by Strickland and Parsons [20] was used to determine on ammonia and nitrite. Water quality characteristics during the experiment period were recorded as followed: pH 7.82 ± 0.23, temperature 27.42 ± 0.34 °C, salinity 25.42 ± 1.28 g L^−1^, DO 5.49 ± 0.62 mg L^−1^, conductivity 41.32 ± 1.25 mS cm^−1^, TDS 22.28 ± 2.87 g L^−1^, total ammonia nitrogen 0.18 ± 0.04 mg L^−1^ and nitrite 0.62 ± 0.13 mg L^−1^.

For breeder preparation, ten ovigerous females of the *E. singaporense* crabs, with the late embryonic stage (dark brown and eyed eggs; 16.8–21.5 g body weight (BW) and 27.7–32.1 mm carapace width (CW)) were gathered by hand at high tide from the mangrove forest in Trang Province, Thailand (7°32′16.68″ N, 99°19′04.44″ E). Within a few hours of harvesting, the gravid female crabs were moved to the Marine Crab Research Laboratory, Rajamangala University of Technology Srivijaya. The females were then placed at 20 L hatching bucket (1 individual per bucket) for incubation of the eggs and larval hatching. Each hatching bucket was filled with 15 L filtered sterilized seawater (salinity 25 g L^−1^, temperature 28 ± 1 °C) and supplied with moderate aeration. No diet was given to the berried females. Feces and unhatched egg deposited at bottom of the hatching bucket were eliminated from the bucket daily, followed by about 10% water exchange. The females were observed every 12 h to monitor the newly hatched larvae. The larvae hatched from four female crabs (*n* = 4) were used to produce early juveniles. The female was removed from the hatching container after had hatched its eggs. The aeration of the water in the hatching tank was stopped, and the active newly hatched larvae (zoea I), which concentrated on the surface of the water, were gathered within 1 h after hatching and transferred to a 500 L fiber nursery tank, which contained 100 L of sterilized seawater.

The zoea I larvae were cultivated in the nursery tank with an initial density of 60–70 larvae L^−1^. Rotifers, without enrichment, were provided directly to the larvae twice daily. When the zoea II stage was reached, mixed live diet (rotifer and newly hatched *Artemia*) were fed to the zoea II larvae, and the volume of water in the nursery tank was increased to 200 L. After the larvae developed into zoea III, the seawater in the nursery tank was raised to 350 L, and only freshly hatched *Artemia* (20–24 h after hatching) were fed to the larvae. The 350 L of seawater in the nursery tank was retained until the larvae entered the stage of crab I, but 30% of the water was exchanged daily. Twice a day, only adult *Artemia* with different densities were supplied as live diet to zoea IV and megalopal larvae, as described in Sudtongkong et al. [21]. Small branches with leaves of pine tree were added as shelters to minimize cannibalism during the megalopal stage. Additionally, sediment and waste deposited at the bottom of the nursery were also siphoned off regularly. After entering the crab I stage, they were moved to culture at a concrete tank (2.0 m width × 4.0 m length × 0.8 m depth), with a density of 1000 crabs per tank. During the early juvenile (crab I) cultivation time, the small branches with leaves of pine trees and seaweed (*Caulerpa* spp.) were used to minimize cannibalism among these crabs. Until reaching the stage of crab III (2 months of age), early juveniles were fed fresh diets such as shrimp meat, fish meat (10% BW). Conditions of seawater in the nursery tank and in the concrete tank were maintained at pH 7–8, salinity 25 g L^−1^, temperature 27–28 °C and DO > 5 mg L^−1^.

Prior to the experiment, the juvenile crabs in the cement tank were acclimated by being fed a commercial juvenile shrimp diet, containing 30% crude protein and 5% crude lipid (Charoen Pokphand Foods PCL., Bangkok, Thailand), for 2 weeks. The experiment conducted used a completely randomized design. When this experiment was begun, 600 early juvenile *E. singaporense* crabs (0.10 ± 0.02 g initial BW and 5.57 ± 0.51 mm initial CW) were randomly captured from the concrete tank, and transferred for individual rearing in 1 L polypropylene plastic cups (9.5 cm diameter × 14.5 cm height). Each rearing cup was covered by a dome lid to prevent the animals escaping and was filled with 200 mL filtered sterilized seawater (salinity 25 g L^−1^, temperature 28 ± 1 °C). Sixty juvenile crabs individually stored in sixty plastic cups were used to evaluate the effect of each diet on their survival and growth. The crabs were fed once daily at 16.00 h. Feces and uneaten diet were removed daily from the rearing cup accompanied by an approximately 80% water exchange. Molts and deaths were recorded on a daily basis. The feeding rate was 10% of the overall BW when started the experiment. During the experimental period, the quantity of daily diet was balanced by BW every two weeks, and the unconsumed diet was removed to calculate the weight of consumed diet (feed intake), according to the method described by Jin et al. [10]. These diets were oven-dried at 50 °C for 12 h to eliminate water before weighing. The research was performed for a 6-week duration. At the end of the experiment, all the crabs were fasted for 24 h prior to sampling. Each individual crab was weighed on a digital microbalance (Ohaus AR2140; Ohaus Corp., Parsippany, NJ, USA) after blotting them dry with a paper towel, and their CW (maximum distance across the carapace) was measured to the nearest 0.01 mm using a digital vernier caliper (Mitutoyo-500; Mitutoyo Corp., Kanagawa, Japan). The digestive tracts from 4−6 crabs each were carefully removed on ice, pooled, packed in 1.5 mL centrifuge tubes, and kept at −20 °C, until being used to evaluate the specific activity of the digestive enzymes. The muscle from the same samples were used for determination of amino acid profiles. The growth performance and feed utilization characteristics of reared crabs at the end of experiment were calculated as follows:

Survival (%) = [Final crab number/initial crab number] × 100(1)

Weight gain (WG, %) = [(Final BW − initial BW)/initial BW] × 100(2)

CW gain (CWG, %) = [(Final CW − initial CW)/initial CW] × 100(3)

Specific growth rate (SGR, % BW day^−1^) = [(ln W_t_ − ln W_0_)/(t − t_0_)] × 100 (4)

where W_t_ = mean weight (g) at day t, W_0_ = mean weight (g) at day t_0_.

Feed conversion ratio (FCR, g feed g gain^−1^) = Dry feed consumed (g)/wet WG (g) (5)

Protein efficiency ratio (PER, g gain g protein^−1^) = Wet WG (g)/protein intake (g) (6)

The frozen digestive tract of the crabs (*n* = 5 pooled sample per treatment) was weighed, rinsed with distilled water, and then extracted in cold distilled water at a ratio of 1:3 (*w/v*). Their homogenates were obtained through a micro-homogenizer, with a 7 mm × 195 mm sawtooth generator probe (THP-220; Omni International, Kennesaw GA, USA) after mixing for 10 s. Centrifugation was performed at 15,000× *g* for 30 min at 4 °C, and the supernatants were collected and kept at −20 °C. All the steps were performed on ice to avoid loss of enzyme activity, and all analyses of protein and enzyme activity were determined within 1 month after extraction. The protein concentration of the crude enzyme extract was determined according to the standard method of Lowry et al. [22], using bovine serum albumin as the protein standard. The conditions and protocols for assaying the digestive enzymes, based on spectrophotometric method, are shown in Table 3. One unit (U) of pepsin-like activity was defined as an increase of 1.0 in absorbance at 280 nm, while 1U of other observed enzymes is defined as the amount that catalyzed the conversion of 1 μmol of substrate per min.

The estimation of the amino acids in the experimental diets and the muscle of the juvenile *E. singaporense* crabs (*n* = 3 pooled sample per treatment) were conducted at the Central Laboratory (Thailand) Company Limited, Songkhla, Thailand. The determination was conducted using the protocol described by Unnikrishnan and Paulraj [17]. The 0.1 g of fresh weight of the diet and muscle of juvenile crabs with 10 mL of 6 N HCl was digested at 110 °C in glass tubes sealed with a nitrogen atmosphere, for 24 h. The amino acid levels of the diets and muscle were determined after acid hydrolysis using an HPLC analyzer (Agilent 1100 LC/MSD Model G1946D; Agilent Technologies Inc., Santa Clara, CA, USA). The amino acid profiles were summarized as EAA and non-essential amino acids (NEAA). The dietary ratios of individual EAA were compared with ΣEAA (A/E ratio).

As noted above, a completely randomized design was adopted; comprising five treatments of 60 crabs each, as the experimental units. Arc sine transformation was applied to percentages prior to analysis. All statistical evaluations were conducted with the Statistical Package for the Social Sciences Version 14 (SPSS Inc., Chicago, IL, USA). One-way ANOVA was used to determine statistically significant differences, and mean comparisons were carried out using Duncan’s multiple range test (*p* < 0.05).

## 3. Results

### 3.1. Survival, Growth Performance and Feed Utilization

Crabs fed this 30% dietary protein showed lower survival than others fed with a higher percentage of protein level (*p* < 0.05). However, WG, CWG, SGR, and FCR did not differ across all five dietary treatments. Significantly higher PER was observed in the *E. singaporense* crabs receiving 45% dietary protein relative to the remaining treatments (Table 4).

### 3.2. Specific Activities of Digestive Enzymes

**Figure 1:**
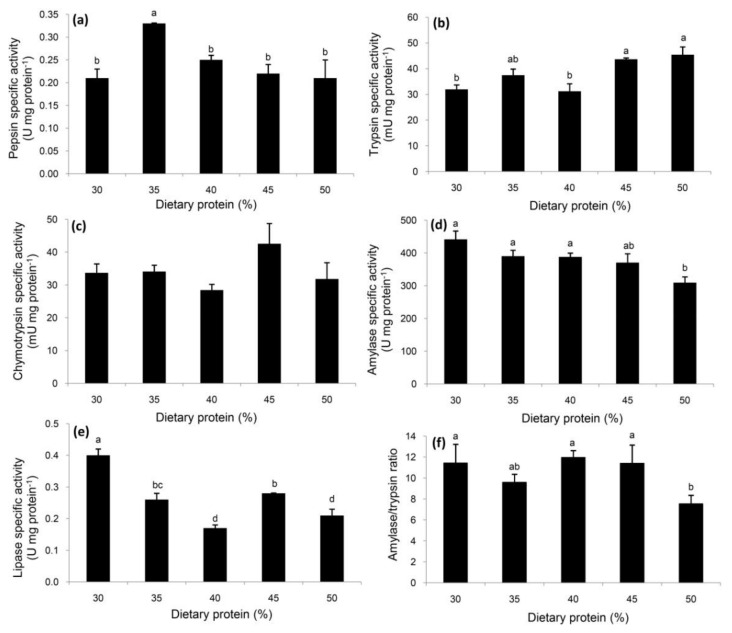
Specific activities of pepsin-like (**a**), trypsin (**b**), chymotrypsin (**c**), amylase (**d**), and lipase (**e**), and the ratio of amylase to trypsin (**f**) in *E. singaporense* crabs fed with various levels of dietary protein. Data are expressed as mean ± SEM (*n* = 5 per treatment). ^a,b,c,d^ Differences between means were tested with Duncan’s multiple range test, and are indicated by different superscripts (*p* < 0.05).

The highest pepsin-like specific activity was observed in the *E. singaporense* crabs receiving 35% dietary protein relative to the remaining treatments (Figure 1a). Trypsin specific activity was highest in the crabs receiving at least 45% protein (Figure 1b), while no differences were observed for chymotrypsin across all five dietary treatments (Figure 1c). Significantly decreased amylase specific activity was observed in the crabs receiving the highest level of protein (50%), relative to the 30%, 35% and 40% treatments, but the difference relative to the 45% protein group was not significant (Figure 1d). Lipase specific activity was highest in the crabs receiving 30% protein, followed by 35% and 45% (Figure 1e). A similar pattern was observed for the activity ratio of amylase to trypsin to that noted for amylase specific activity (Figure 1d,f).

### 3.3. Muscle Amino Acid Composition

Generally, the crabs receiving 45% protein diets had superior EAA profiles, although some amino acids gave moderate values, with those receiving 40% protein having the next highest level of EAA. The pattern was reversed for the NEAA profiles. The ΣEAA/ΣNEAA ratio was highest in crabs receiving 45% protein diets, followed by those receiving 50%, 30% or 35%, and 40%, respectively. Arginine and lysine were the major amino acids, based on A/E ratio, followed by leucine, relative to the remaining amino acids (Table 5).

## 4. Discussion

Generally, the survival of juvenile crabs is in a wide range of 0 to 100% [10,17]. In the current study, relatively high survival was observed, and the values were within the range of 34 to 88%, depending on the dietary protein levels administered. Crabs receiving the lowest protein level had significantly decreased survival relative to the other treatments. This finding is in agreement with the significant effect of dietary protein levels noted on the survival of juvenile giant mud crab (*Scylla serrata*), where a diet containing 15% protein resulted in 100% mortality relative to diets containing 20% protein (87.5% survival) or 25–55% protein (100% survival) [17]. However, no significant effects on survival were observed for dietary protein levels of 30–55% or 25–40% in gazami crab (*Portunus trituberculatus*) [10] and whiteleg shrimp (*Litopenaeus vannamei*) [30], respectively. It should be noted that the effect on dietary protein level on survival is associated with other factors, such as species, feeding habit and developmental stages.

The feeding trial for optimizing the dietary protein levels in various juvenile crab species was conducted in a range of five to eight weeks [9,10,17,31]. In the current study, a feeding trial ensured a 6-week period to individual crab in each dietary treatment, avoiding any errors associated with asynchrony in the first moulting of juvenile crabs, from which the feeding trial commenced [17]. In addition, preliminary study in *E. singaporense* indicates low feed consumption and high mortality rate when the experiment was prolonged. It may be caused by the increased stress of crabs that prolongs reared in small containers. Thus, an extended feeding period of more than six weeks may lead to an error in the protein requirement experiment of this crab.

A microbound diet was used to rear the juvenile crabs in the current study, since it is more suitable for juvenile crustaceans, relative to a microencapsulated diet [11,32]. Based on growth performance, no differences were observed across the five dietary treatments, suggesting that the crabs were able to compensate growth over the period studied, while significantly improved PER was only found in crabs receiving the 45% protein level. No differences in WG were observed across five dietary treatment and the values were relatively low since they molted once or twice within the six weeks trial (from 0.1 g initial BW until 0.15 g final BW). Relatively low growth performance (from 1.03–1.08 g initial BW, until 1.32–1.50 g final BW) has been reported in Chinese hairy crab (*Eriocheir sinensis*) after feeding with dietary protein levels of 29.8–54.8% over a 5-week trial [9]. It is possible that the biology and adaptation of both species to dietary intake are similar, or the environmental conditions for rearing are inappropriate that may have contributed to the lower WG. The optimal protein level from the current study is within the range of dietary protein previously recommended for mariculture of crustaceans of between 30 and 55% [9,10,15,16,17], and is similar to the dietary protein levels typically used in formulated diets for juvenile crustaceans, namely 46% protein for giant tiger prawn (*Penaeus monodon*) [33] and 47.9–50.8% protein for giant freshwater prawn (*Macrobrachium rosenbergii*) [34].

Some nutritional changes were observed through the activities of all the protein-digesting enzymes, except for chymotrypsin, suggesting the modulation of protein catabolism due to different dietary protein levels. The presence of pepsin-like activity has been reported in adult lobsters and crabs [35,36], as well as in the early developmental stages of *E. singaporense* crab [21], spider crab [23], and mud crab, *Scylla paramamosain* [37]. The highest specific activity in crabs receiving 35% protein might be due to the relatively high amount of preferably aromatic amino acids in their diet, such as phenylalanine, tryptophan, and tyrosine, which are most efficient in cleaving peptide bonds [38]. Although the crabs were fasted prior to sampling, the post-prandial expression of pepsin-like might be rhythmic and available for the subsequent digestion of protein. However, the role of this enzyme remains unclear, and it appears to be induced by physiological processes [36]. The colorimetric determination of pepsin-like in the current study was based on the hydrolysis of non-specific substrate, hemoglobin, so that the detection of activity is likely an expression of other acidic proteases, such as lysosomal cathepsin D, that are involved in the extracellular and intracellular digestion processes [39]. Enzyme inhibitors should be used to identify the type of enzymes that are in the extracts. Previous publications have tended to investigate proteolytic activity in alkaline conditions, since alkaline-type proteases might play a major role in protein homeostasis [23,40]. In *E. singaporense* crabs, trypsin was noted to be more sensitive to ontogenic changes than chymotrypsin [21], and this agrees with the results of the dietary protein levels administered in the current study, where an abrupt increase in trypsin activity was observed in crabs receiving 45 and 50% protein, suggesting a high catabolism of protein particularly in the intestinal section. This finding is in agreement with the results of Péres et al. [41] and Krogdahl et al. [42], who stated that increased dietary protein levels can stimulate the pancreas to secret trypsin.

A significant reduction in amylase specific activity was observed in the crabs receiving the highest amount of protein. This trend agrees with the amount of NFE in the experimental diets, ranging from 41.67 to 15.68%. However, the lack of effects from amylase in a wide range of NFE (41.67 to 22.73%) suggests that there was comparatively little carbohydrate utilization and that it plays a minor role in *E. singaporense* crabs. The A/T ratio describes the utilization of carbohydrate per amount of protein [43], and significant changes in this ratio indicate an imbalance in the proportional use of those nutritional components in crabs receiving 50% protein.

Lipase plays an important role in digesting dietary lipids as an energy reserve [44,45]. The highest lipase specific activity was observed in the crabs receiving the lowest level of dietary protein, but the values did not reflect the fact that there were similar lipid contents in all the experimental diets, suggesting some physiological changes due to the variation in the dietary protein level. In *E. singaporense* crabs, a protein-sparing effect by lipid-digesting enzymes was observed by Sudtongkong et al. [21], so it is possible that the lipid digestion increased when the protein digestion via pepsin-like and trypsin decreased.

The highest efficiency of muscle protein retention was observed in animals fed by optimal protein level, relative to suboptimal or excessive levels [46]. The crabs receiving the 45% protein diet had a superior muscle EAA content and a superior ΣEAA/ΣNEAA ratio, relative to the remaining treatments, and this finding is in good agreement with the PER values of this treatment. Based on the Pearson correlation analysis, a positively significant relationship was observed between the A/E ratios from experimental diets and muscle (*p* < 0.05, R^2^ = 0.736), indicating the crabs received the dietary EAA to generate muscle. In juvenile giant mud crab, similarity between A/E ratios from experimental diet and whole carcass has been reported [17]. This index is a useful tool for evaluating amino acid balance from dietary protein intake. A high amount of arginine and lysine in muscle, followed by leucine, relative to the remaining amino acids, indicated the major types of EAA for *E. singaporense*. This finding is in agreement with the major EAAs found in juvenile stage of spider crab [46]. However, a variation in A/E ratios across various publications occurred, due to the differences in dietary protein level, developmental stage, and animal species [47,48]. Generally, protein breakdown from critical tissues occurs in animals receiving low amounts of protein, which cannot meet bodily protein requirements [7], so that significantly decreased PER, EAA, and ΣEAA/ΣNEAA ratios were observed in crabs receiving 30 to 40% protein diets. On the other hand, an excessive amount of dietary protein can cause significant reduction in growth performance [49], or does not improve the SGR or protein productive value significantly [10]. A high amount of dietary protein intake can lead to the production and accumulation of ammonia in the hemolymph, which negatively influences metabolic processes, particularly osmotic pressure balance and oxygen transport [50], as well as causing toxicity, due to the accumulation of free amino acids [10]. Therefore, energy would be used to excrete nitrogen wastes; nitrogen loss increased as the dietary protein increased [30], leading to growth retardation [13].

## 5. Conclusions

Based on the survival, growth performance, feed utilization, digestive enzyme specific activities, and muscle amino acid composition, the optimal dietary protein requirement for juvenile *E. singaporense* crabs might be 45%. This level is similar to previous reports in other crab species, and could be employed in preparing artificial diets for this species. However, other mariculture circumstances, namely crab stages, rearing conditions, and types of diet, might produce different results, and the effect of those variables should be further investigated.

## Figures and Tables

**Table 1:** Formulations and proximate chemical compositions (% of fresh weight) of the experimental diets. The compositions were values from triplicate analyses.

Ingredient and Composition	Experimental Diet (% CP)				
	30	35	40	45	50
Ingredient					
Fish meal	16.75	21.76	26.76	31.76	36.76
Shrimp meal	5	5	5	5	5
Soybean meal	20	20	20	20	20
Wheat gluten meal	12.09	14.92	17.77	20.61	23.46
Fish oil	2.56	2.33	2.11	1.88	1.66
Soybean oil	2.56	2.33	2.11	1.88	1.66
Dextrin	36.24	28.08	19.89	11.74	3.54
Soy lecithin	1	1	1	1	1
Vitamin premix ^a^	0.8	0.8	0.8	0.8	0.8
Mineral premix ^b^	3	3	3	3	3
Cellulose	0	0.78	1.56	2.33	3.12
Composition					
Moisture	10.47 ± 0.21	10.06 ± 0.10	12.61 ± 0.13	8.62 ± 0.20	10.06 ± 0.19
CP	30.71 ± 0.19	35.84 ± 0.21	38.64 ± 0.24	46.34 ± 0.23	50.49 ± 0.19
Crude lipid	6.95 ± 0.18	6.81 ± 0.25	6.41 ± 0.19	7.49 ± 0.17	7.12 ± 0.20
Ash	8.00 ± 0.11	9.74 ± 0.21	10.76 ± 0.18	12.71 ± 0.12	13.99 ± 0.17
Crude fiber	2.20 ± 0.12	2.00 ± 0.15	2.09 ± 0.13	2.11 ± 0.17	2.66 ± 0.10
NFE	41.67 ± 0.15	35.55 ± 0.56	29.49 ± 0.37	22.73 ± 0.41	15.68 ± 0.16
GE (kcal kg^−1^)	4175 ± 10	4189 ± 13	4064 ± 13	4321 ± 5	4252 ± 28

CP, crude protein; NFE, nitrogen-free extract; GE, gross energy. Data are expressed as mean ± SEM. ^a^ Vitamin premix, 1 kg contained 12,000 U vitamin A, 2000 U vitamin D, 3000 mg vitamin E, 20 mg vitamin K, 100 mg thiamine HCl, 150 mg riboflavin, 500 mg niacin, 500 mg *D*-pantothenic acid, 25 mg pyridoxine HCl, 1 mg biotin, 5 mg folic acid, 125 mg vitamin B_12_, 1000 mg ascorbic acid, 1500 mg choline and 1 mg inositol. ^b^ Mineral premix, 1 kg contained sodium 3 g chloride, 1 g potassium chloride, 1.4 g magnesium sulphate, 0.2 g ferric citrate, 0.25 g manganese sulphate, 0.01 g potassium iodide, 0.13 g zinc carbonate, 0.01 g copper sulphate and 6 g dicalcium phosphate.

**Table 2:** Amino acid contents (% of fresh weight) in experimental diets for rearing *E. singaporense* crabs. The compositions were values from triplicate analyses.

Amino Acid	Experimental Diet (% CP)					A/E Ratio
	30	35	40	45	50	
EAA						
Arginine	5.75 ± 0.02	6.25 ± 0.08	4.78 ± 0.06	5.84 ± 0.09	4.20 ± 0.04	0.20
Histidine	0.79 ± 0.07	0.96 ± 0.09	0.76 ± 0.05	1.08 ± 0.04	0.84 ± 0.06	0.03
Isoleucine	1.44 ± 0.06	1.52 ± 0.08	1.27 ± 0.03	1.98 ± 0.04	1.46 ± 0.06	0.06
Leucine	2.77 ± 0.02	3.07 ± 0.04	2.74 ± 0.08	3.42 ± 0.10	3.24 ± 0.10	0.12
Lysine	4.77 ± 0.04	4.83 ± 0.09	4.02 ± 0.10	5.20 ± 0.12	4.29 ± 0.08	0.18
Methionine	5.57 ± 0.04	5.61 ± 0.02	4.90 ± 0.12	5.41 ± 0.05	4.41 ± 0.09	0.20
Phenylalanine	1.34 ± 0.08	1.56 ± 0.07	1.34 ± 0.09	1.67 ± 0.05	1.68 ± 0.12	0.06
Threonine	1.97 ± 0.09	2.09 ± 0.07	1.59 ± 0.06	2.25 ± 0.07	1.69 ± 0.06	0.07
Tryptophan	0.60 ± 0.02	0.63 ± 0.02	0.60 ± 0.02	0.40 ± 0.01	0.77 ± 0.02	0.02
Valine	1.65 ± 0.04	1.65 ± 0.07	1.46 ± 0.03	2.19 ± 0.05	1.73 ± 0.04	0.07
ΣEAA	26.65 ± 0.17	28.17 ± 0.13	23.46 ± 0.14	29.44 ± 0.09	24.3 ± 0.12	
NEAA						
Alanine	2.57 ± 0.06	2.28 ± 0.09	2.28 ± 0.04	2.96 ± 0.06	2.83 ± 0.08	
Aspartic acid	2.20 ± 0.09	2.15 ± 0.06	1.54 ± 0.10	2.66 ± 0.12	1.52 ± 0.12	
Asparagine	nd	nd	nd	nd	nd	
Cysteine	nd	nd	nd	nd	nd	
Cystine	0.03 ± 0.01	0.41 ± 0.02	0.13 ± 0.01	0.31 ± 0.02	0.27 ± 0.02	
Glutamic acid	4.85 ± 0.08	4.50 ± 0.09	3.84 ± 0.07	5.42 ± 0.10	3.07 ± 0.11	
Glutamine	0.98 ± 0.07	1.41 ± 0.06	0.80 ± 0.06	1.23 ± 0.10	1.49 ± 0.05	
Glycine	2.80 ± 0.08	2.85 ± 0.07	2.55 ± 0.08	3.50 ± 0.09	3.05 ± 0.09	
Proline	4.11 ± 0.03	4.50 ± 0.02	3.72 ± 0.04	5.52 ± 0.02	3.95 ± 0.03	
Hydroxyproline	0.33 ± 0.05	0.29 ± 0.04	0.23 ± 0.02	0.46 ± 0.03	0.30 ± 0.01	
Serine	2.25 ± 0.07	2.21 ± 0.06	1.69 ± 0.04	2.45 ± 0.09	1.80 ± 0.07	
Tyrosine	0.57 ± 0.01	1.00 ± 0.03	0.69 ± 0.02	1.02 ± 0.03	0.90 ± 0.03	
ΣNEAA	20.69 ± 0.14	21.6 ± 0.21	17.47 ± 0.17	25.53 ± 0.27	19.18 ± 0.25	
ΣEAA/ΣNEAA	1.29 ± 0.01	1.31 ± 0.02	1.34 ± 0.02	1.15 ± 0.01	1.27 ± 0.01	

CP, crude protein; EAA, essential amino acids; NEAA, non-essential amino acids; A/E ratio, individual EAA/ΣEAA; nd, not detected. Data are expressed as mean ± SEM.

**Table 3:** Protocols used for determination of digestive enzyme specific activity in *E. singaporense*.

Enzyme	Condition *	Substrate **	Product	Absorbance
Pepsin-like	pH 2 at 37 °C [23]	Hemoglobin	-	280 nm
Trypsin	pH 9 at 60 °C [24]	BAPNA	*p*-Nitroanilide	410 nm
Chymotrypsin	pH 9 at 40 °C [24]	SAPNA	*p*-Nitroanilide	410 nm
Amylase	pH 7 at 50 °C [24]	Starch	Maltose	540 nm
Lipase	pH 8 at 60 °C [25]	*p*-NPP	*p*-Nitrophenol	410 nm

* The optimal conditions were chosen from previous reports in spider crab, *Maja brachydactyla* [23]; blue swimming crab, *Portunus pelagicus* [24]; and green crab, *Carcinus mediterraneus* [25]. ** Determination of pepsin-like [26], trypsin [27], chymotrypsin [27], amylase [28] and lipase [29] activities were conducted following the previous publications. BAPNA, benzoyl-*L*-Arg-*p*-nitroanilide; SAPNA, *N*-succinyl-Ala-Ala-Pro-Phe-*p*-nitroanilide; *p*-NPP, *p*-nitrophenyl palmitate.

**Table 4:** Survival, growth performance, and feed utilization of *E. singaporense* crabs fed with experimental diets varying in protein contents. The observed parameters were recorded at the end of the six-week experiment.

Parameter	Experimental Diet (% CP)				
	30	35	40	45	50
Survival (%)	34.43 ± 14.90 ^b^	75.03 ± 5.91 ^a^	78.12 ± 10.87 ^a^	87.5 ± 3.34 ^a^	78.13 ± 6.95 ^a^
WG (%)	29.58 ± 10.22	31.15 ± 10.20	32.42 ± 9.04	46.67 ± 7.70	33.33 ± 8.33
CWG (%)	10.05 ± 2.94	12.69 ± 4.83	10.49 ± 3.19	21.74 ± 4.06	11.69 ± 3.70
SGR (% BW day^−1^)	0.41 ± 0.14	0.43 ± 0.10	0.47 ± 0.21	0.68 ± 0.09	0.36 ± 0.12
FCR (g feed g gain^−1^)	1.94 ± 0.33	1.67 ± 0.31	1.66 ± 0.32	1.17 ± 0.06	2.05 ± 0.53
PER (g gain g protein^−1^)	0.17 ± 0.03 ^b^	0.23 ± 0.08 ^b^	0.26 ± 0.06 ^b^	0.54 ± 0.09 ^a^	0.29 ± 0.04 ^b^

CP, crude protein; WG, weight gain; CWG, carapace width gain; SGR, specific growth rate; BW, body weight; FCR, feed conversion ratio, PER, protein efficiency ratio. Data are expressed as mean ± SEM (*n* = 60 per treatment). Differences between means were tested with Duncan’s multiple range test. Different superscripts in the same row indicate a significant difference (*p* < 0.05).

**Table 5:** Amino acid contents (% of fresh weight) in the muscle of *E. singaporense* crabs. The analysis was performed at the end of the experiment.

Amino Acid	Experimental Diet (% CP)					A/E Ratio
	30	35	40	45	50	
EAA						
Arginine	1.41 ± 0.01 ^c^	1.79 ± 0.01 ^b^	1.91 ± 0.01 ^a^	1.92 ± 0.01 ^a^	1.34 ± 0.01 ^d^	0.24
Histidine	0.24 ± 0.01 ^c^	0.26 ± 0.01 ^d^	0.35 ± 0.01 ^b^	0.37 ± 0.01 ^a^	0.28 ± 0.01 ^c^	0.04
Isoleucine	0.38 ± 0.01 ^e^	0.48 ± 0.01 ^c^	0.56 ± 0.01 ^a^	0.55 ± 0.01 ^b^	0.44 ± 0.01 ^d^	0.07
Leucine	0.68 ± 0.01 ^d^	0.83 ± 0.01 ^c^	1.07 ± 0.01 ^a^	1.04 ± 0.01 ^b^	0.83 ± 0.01 ^c^	0.13
Lysine	1.11 ± 0.01 ^d^	1.30 ± 0.01 ^c^	1.80 ± 0.01 ^a^	1.81 ± 0.01 ^a^	1.59 ± 0.01 ^b^	0.22
Methionine	0.35 ± 0.01 ^d^	0.39 ± 0.01 ^b^	0.36 ± 0.01 ^c^	0.47 ± 0.01 ^a^	0.36 ± 0.01 ^c^	0.06
Phenylalanine	0.40 ± 0.01 ^e^	0.46 ± 0.01 ^d^	0.60 ± 0.01 ^b^	0.64 ± 0.01 ^a^	0.49 ± 0.01 ^c^	0.08
Threonine	0.36 ± 0.01 ^d^	0.45 ± 0.01 ^b^	0.55 ± 0.01 ^a^	0.55 ± 0.01 ^a^	0.43 ± 0.01 ^c^	0.07
Tryptophan	0.15 ± 0.01 ^d^	0.17 ± 0.01 ^c^	0.19 ± 0.01 ^a^	0.19 ± 0.01 ^a^	0.18 ± 0.01 ^b^	0.03
Valine	0.39 ± 0.01 ^d^	0.50 ± 0.01 ^b^	0.58 ± 0.01 ^a^	0.49 ± 0.01 ^c^	0.39 ± 0.01 ^d^	0.07
ΣEAA	5.48 ± 0.01 ^e^	6.62 ± 0.01 ^c^	7.97 ± 0.02 ^b^	8.03 ± 0.01 ^a^	6.33 ± 0.01 ^d^	
NEAA						
Alanine	0.57 ± 0.01 ^d^	0.75 ± 0.01 ^b^	0.86 ± 0.01 ^a^	0.62 ± 0.01 ^c^	0.55 ± 0.01 ^e^	
Aspartic acid	1.01 ± 0.01 ^e^	1.51 ± 0.01 ^c^	1.74 ± 0.01 ^a^	1.70 ± 0.01 ^b^	1.43 ± 0.01 ^d^	
Cysteine	0.03 ± 0.01	0.02 ± 0.01	nd	nd	nd	
Cystine	0.08 ± 0.01 ^d^	0.10 ± 0.01 ^c^	0.11 ± 0.01 ^b^	0.15 ± 0.01 ^a^	0.11 ± 0.01 ^b^	
Glutamic acid	1.11 ± 0.01 ^e^	1.56 ± 0.01 ^d^	1.77 ± 0.01 ^b^	1.86 ± 0.10 ^a^	1.59 ± 0.01 ^c^	
Glutamine	1.17 ± 0.01 ^b^	0.91 ± 0.01 ^c^	1.47 ± 0.01 ^a^	1.19 ± 0.01 ^b^	0.99 ± 0.01 ^c^	
Glycine	0.99 ± 0.01 ^e^	1.29 ± 0.01 ^c^	1.60 ± 0.01 ^a^	1.36 ± 0.01 ^b^	1.09 ± 0.01 ^d^	
Proline	0.65 ± 0.01 ^c^	0.65 ± 0.01 ^c^	0.91 ± 0.01 ^a^	0.71 ± 0.01 ^b^	0.55 ± 0.01 ^d^	
Serine	0.32 ± 0.01 ^e^	0.42 ± 0.01 ^c^	0.52 ± 0.01 ^a^	0.48 ± 0.01 ^b^	0.38 ± 0.01 ^d^	
Tyrosine	0.39 ± 0.01 ^d^	0.44 ± 0.01 ^c^	0.53 ± 0.01 ^b^	0.60 ± 0.01 ^a^	0.45 ± 0.01 ^c^	
ΣNEAA	6.33 ± 0.01 ^e^	7.66 ± 0.01 ^c^	9.52 ± 0.01 ^a^	8.68 ± 0.10 ^b^	7.13 ± 0.01 ^d^	
ΣEAA/ΣNEAA	0.87 ± 0.01 ^c^	0.86 ± 0.01 ^c^	0.84 ± 0.01 ^d^	0.92 ± 0.01 ^a^	0.89 ± 0.01 ^b^	

CP, crude protein; EAA, essential amino acids; NEAA, non-essential amino acids; A/E ratio, individual EAA/ΣEAA; nd, not detected. Data are expressed as mean ± SEM (*n* = three per treatment). Differences between means were tested with Duncan’s multiple range test. ^a,b,c,d,e^ Different superscripts in the same row indicate a significant difference (*p* < 0.05).
